# Multi-omics analysis reveals cuproptosis and mitochondria-based signature for assessing prognosis and immune landscape in osteosarcoma

**DOI:** 10.3389/fimmu.2023.1280945

**Published:** 2024-01-05

**Authors:** Chenguang Jia, Mei Liu, Liming Yao, Fangchao Zhao, Shuren Liu, Zhuo Li, Yongtai Han

**Affiliations:** ^1^ Department of Osteonecrosis and Hip Surgery, The Third Hospital of Hebei Medical University, Shijiazhuang, China; ^2^ Department of Orthopedics, Hebei Chest Hospital, Shijiazhuang, China; ^3^ Molecular Biology Laboratory, Hebei Chest Hospital, Shijiazhuang, China; ^4^ Department of Thoracic Surgery, The Second Hospital of Hebei Medical University, Shijiazhuang, China

**Keywords:** osteosarcoma, cuproptosis, mitochondria dysfunction, prognostic biomarker heterogeneity, stemness

## Abstract

**Background:**

Osteosarcoma (OSA), the most common primary mesenchymal bone tumor, is a health threat to children and adolescents with a dismal prognosis. While cuproptosis and mitochondria dysfunction have been demonstrated to exert a crucial role in tumor progression and development, the mechanisms by which they are regulated in OSA still await clarification.

**Methods:**

Two independent OSA cohorts containing transcriptome data and clinical information were collected from public databases. The heterogeneity of OSA were evaluated by single cell RNA (scRNA) analysis. To identify a newly molecular subtype, unsupervised consensus clustering was conducted. Cox relevant regression methods were utilized to establish a prognostic gene signature. Wet lab experiments were performed to confirm the effect of model gene in OSA cells.

**Results:**

We determined 30 distinct cell clusters and assessed OSA heterogeneity and stemness scRNA analysis. Then, univariate Cox analysis identified 24 candidate genes which were greatly associated with the prognosis of OSA. Based on these prognostic genes, we obtained two molecular subgroups. After conducting step Cox regression, three model genes were selected to construct a signature showing a favorable performance to forecast clinical outcome. Our proposed signature could also evaluate the response to chemotherapy and immunotherapy of OSA cases.

**Conclusion:**

We generated a novel risk model based on cuproptosis and mitochondria-related genes in OSA with powerful predictive ability in prognosis and immune landscape.

## Introduction

Osteosarcoma (OSA) is one of the most predominant primary neoplasms of malignancy in childhood while also being an important cause of tumor-related death in adolescence. Early OSA treatment is mainly limited to local surgical resection treatment, but therapeutic effects have been dismal (1). With the popularization of the four major chemotherapeutic agents and the development of neoadjuvant therapies for OSA, the survival rate of patients has increased substantially (2). The most significant factors preventing OSA patients from surviving longer today are tumor recurrence and metastasis. Highly malignant OSA exhibits remarkable early lung metastases that progress rapidly in the absence of treatment (3). Consequently, there is an urgent need to develop novel biomarkers to block the OSA metastasis in order to boost the survival prognosis of patients.

Mitochondria play a crucial role in cancer development by being involved in energy production, cell metabolism, and cell signaling. They are essential not only for ATP synthesis but also for lipid and nucleic acid metabolism as well as tumor development and metastasis (4). Mitochondria mediate crosstalk between tumor cells and their microenvironment by activating, interacting with, and regulating cells within the tumor microenvironment (TME). Mitochondrial metabolism influences multiple processes that underpin tumor progression such as proliferation of transformed cells, resistance to apoptosis, and ability to invade surrounding tissues (5). In addition to central bioenergetic functions, mitochondria provide building blocks for tumor anabolism while controlling redox and calcium homeostasis as well as participating in transcriptional regulation and governing cell death (6).

Metal ions are essential micronutrients for the human body, but insufficient or excessive levels of metals will trigger cell death. Copper is one of the heavy metal ions having an important role in biological processes such as mitochondrial respiration as well as antioxidation (7). Tsvetkov et al. identified and defined a novel regulatory cell death (RCD) modality, cuproptosis, which induces cell death through copper ion targeting of tricarboxylic acid (TCA) cycle proteins. Although organisms have a physiological requirement for copper and copper deficiency disrupts the function of copper-binding enzymes, excessive copper can also cause cell death. Excess copper accumulation triggers the destruction of iron-sulfur cofactors, initiates copper-driven Fenton reactions, and generates destructive ROS, leading to oxidative stress and oxidative damage to tumor tissues. Copper homeostasis is closely related to tumor cell proliferation, angiogenesis and metastasis. A genome-wide CRISPR-Cas9 technology screened specific metabolic pathways that mediate copper death (8). The researchers employed two copper ion carriers, Elisimo and DTC, to separately treat human ovarian cancer cells, and determined 10 genes that may be associated with copper death from the common interval between the two. Previous studies have demonstrated that Elisimo can directly target FDX1 gene which encodes a reductase reducing Cu^2+^ to the more toxic Cu^+^. Similar to the role exerted by FDX1, LIAS, LIPT1 and DLD are also involved in protein lipid acylation metabolism (8). Recent studies have demonstrated a strong link between copper-induced cell death and tumor (9, 10), suggesting that cuproptosis plays a key role in cancer progression, but its role in OS is still unknown.

In this project, we developed a robust risk model based on mitochondria and cuproptosis-related genes. This model could assess prognosis of OSA cases and predict immune landscape and drug response, which in turn provide valuable option for clinical decision-making.

## Materials and methods

### Data collection and arrangement

The transcriptome expression profiling and clinical data of 84 OSA cases were obtained from the TARGET database. Another independent OSA cohort with 53 OSA cases (GSE21257) was accessed from the GEO database. The mRNA expression data of OSA and normal samples in GSE99671 was downloaded for identifying differentially expressed genes (DEGs). The mitochondria-related genes (MRGs) were accessed from the MitoCarta3.0 database. Cuproptosis-related genes (CRGs) were identified based on previous reports (8).

### Function enrichment analysis

The Gene Ontology (GO) and the Kyoto Encyclopedia of Genes and Genomes (KEGG) enrichment were employed to detect the function and pathway of candidate genes through the ‘ggplot2’ package (11). The gene set variation analysis (GSVA) was employed by ‘GSVA’ package (12).

### Single-cell RNA (scRNA) analysis

We obtained the scRNA dataset of OSA (GSE152048) from GEO the database. The normalization and data quality control of scRNA data were conducted by ‘Seurat’ package (13). The dimension reduction was performed by ‘RunPCA’ method. The percentage of CRGs or MRGs in each cell can be obtained by importing CRGs or MRGs through the ‘PercentageFeatureSet’ function. The ‘FindMarkers’ algorithm was employed to screen DEGs between different groups of OSA cells. Moreover, we unearthed the cell communication through R package ‘iTalk’.

### Determination of a novel molecular subgroup

To identify a novel molecular subgroup, unsupervised cluster analysis was conducted by ‘ConsensusClusterPlus’ package (14). The ‘K-Means’ function was employed and ‘euclidean’ was used as a measure of distance, accompanied by resampling of 80% of the items and 1000 replications. The optimal k value was generated based on the proportion of ambiguous clustering (PAC).

### Development of the prognostic risk model

The candidate genes from scRNA analysis were included into univariate Cox analysis to selected prognostic genes for model construction. Next, the dimension reduction of genes was conducted by LASSO regression. Finally, the prognostic risk model was built up by multivariate Cox regression based on the following formula: $\text{Risk value}=\sum_{i=1}^{n}coef_{i}*expression\;level\;of\;gene_{i}$ . The coef represents the coefficient of each gene calculated by multivariate Cox analyses. The OSA patients were divided into high- and low-risk groups according to the median risk value.

### Prediction of immunogenomic landscape

CIBERSORT is a gene expression-based deconvolution algorithm that has been used to assess the percentage of immunocyte infiltration in patients. It was applied to reveal the correlation between model genes and the infiltration level of immune cells in samples (15). The immune activity of OSA samples in two subgroups was assessed by single sample gene set enrichment analysis (ssGSEA) method (16).

### Prediction of drug sensitivity and immunotherapy

The drug response of OSA cases were analyzed by the ‘pRRophetic’ package (17) generating IC50 of different drugs. In addition, we detected the interaction of model genes and three chemotherapy drugs (Adriamycin, Ifosfamide, Methotrexate) by MOE software which could conduct molecular docking analysis. TIDE (http://tide.dfci.harvard.edu/) is a computational tool for evaluating the possibilities for cancer immune escape based on the gene expression profiling of patients.

### Cell culture and cell transfection

The osteoblast cell line (hFOB1.19) and human OSA cell lines (HOS, Saos-2 and 143B) were purchased from the Procell Life Science&Technology Co.,Ltd. (Wuhan, China). The cell lines were cultured in Dulbecco’s modified Eagle’s medium (DMEM) containing 10% fetal bovine serum (FBS) and 1% penicillin/streptomycin and were grown in an incubator at 37°C with 5% CO_2_. The silencing RNA against PTN (si-PTN) was synthesized and purchased from TsingkeBiotechnology Co.,Ltd. (Beijing, China). The sequence of si-PTN is shown in **Supplementary Table 1**. Lipofectamine 3000 (Invitrogen) was used for cell transfection.

### RNA extraction and quantitative real-time polymerase chain reaction (qRT-PCR)

Total RNA was extracted from cell samples by RNAeasy Reagent (Vazyme, China). Total RNA was amplified by qRT-PCR by SYBR qPCR Mix (Vazyme, China). The primer of were synthesized by TsingkeBiotechnology Co.,Ltd. (Beijing, China). The primer sequences are listed in **Supplementary Table 1**. All samples were normalized to GAPDH.

### 5-ethynyl-2′ -deoxyuridine (EdU) assay

The cells were plated in 96-well plates (5,000 cells per well) with DMEM containing 10% FBS for 24 h. Following incubation in 50 mM EdU reagent for 2 h, cells were fixed with 4% paraformaldehyde, permeated by 0.5% Triton X-100, and stained with Apollo reagent for 30 min. Nuclei were stained with Hoechst 33342, and the cells were visualized under a fluorescence microscope.

### Cell migration assay

A Transwell insert with 8 mm pores (Millipore) was utilized to evaluate the OSA cell migration. A total of 10000 cells was cultured in the upper chamber with 200 mL of serum-free DMEM, and 500 mL of medium containing 10% FBS was added to the lower chamber. After 24 h incubation, the migrated cells were fixed with 4% paraformaldehyde and stained by 0.1% crystal violet for 20 min.

### Statistical analysis

Bioinformatics analyses were performed using R software. ANOVA analysis was employed by GraphPad Prism (version 9.0). P values< 0.05 denoted statistically significant differences.

## Results

### Establishment of single-cell transcriptome atlas of OSA

**Figure 1:**
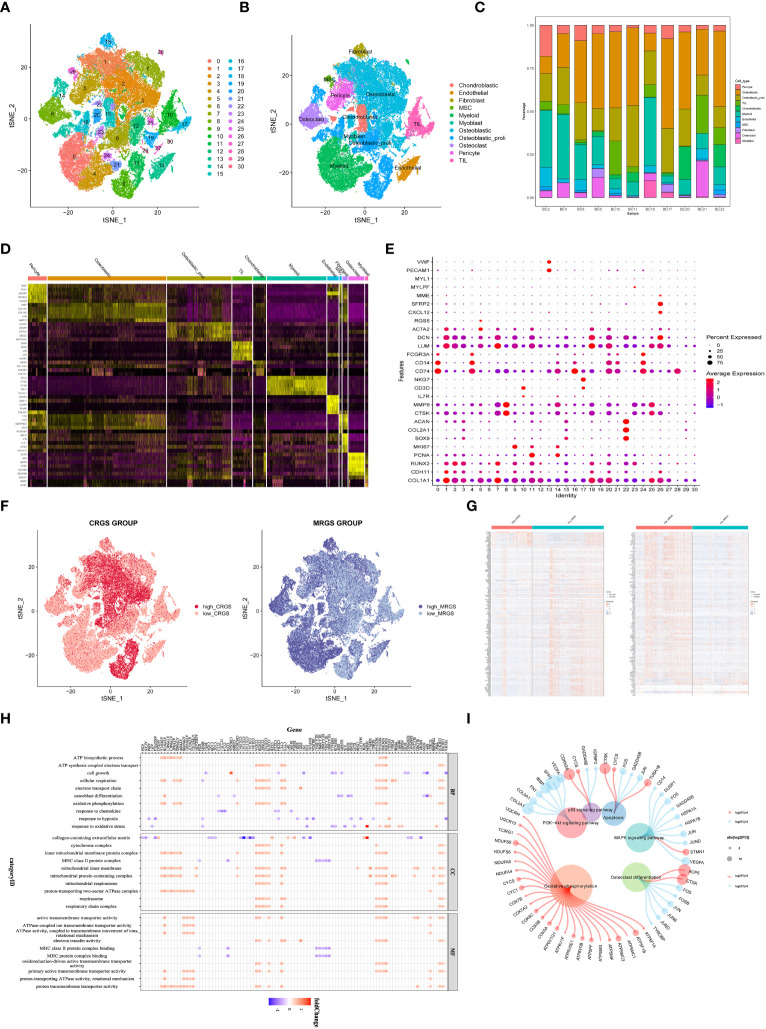
Identification of cuproptosis and mitochondria-related genes according to single cell sequencing analysis in OSA. **(A)** Dimensionality reduction and cluster analysis. **(B)** tSNE clustering of different cell types. **(C)** Distribution of 11 cell types. **(D)** Heatmap showing each cluster and corresponding gene markers. **(E)** All subclusters were annotated based on the composition of the marker. **(F)** The percentage of cuproptosis and mitochondria-related genes in each cell. The cells were divided into high- and low-cuproptosis or mitochondria cells. **(G)** Heatmap displaying DEGs between high- and low-cuproptosis or mitochondria cells. **(H, I)** GO and KEGG enrichment analyses.

We collected adult OSA samples from 11 donors and generated a single-cell transcriptome atlas of OSA cells. Using unsupervised t-SNE clustering and principal component analysis (PCA) for dimension reduction, we identified 30 distinct cell clusters (**Figure 1A**). By leveraging a reference dataset from the Human Primary Cell Atlas, we successfully annotated these cells into 11 different cell types (**Figure 1B**). Additionally, **Figure 1C** illustrates the distribution of these cell clusters across each sample.

Further analysis of gene expression profiles revealed distinct patterns among the 11 cell clusters. We determined the top five marker genes specific to each cell cluster and presented their expression levels through a heat map visualization. Moreover, a dot plot was generated to visualize the expression levels of cell type-specific marker genes (**Figures 1D, E**). Next, 19 CRGs were input by the ‘PercentageFeatureSet’ algorithm, and the percentage of CRGs in each cell was generated. The cells were divided into low and high cuproptosis cells by their median CRG proportion and were defined as low and high CRG score (CRGS) groups. Similarly, we obtained two MRG score (MRGS) groups through the above method (**Figure 1F**). A total of 247 DEGs were obtained between low and high CRG score (CRGS) groups. Also, we screened out 710 DEGs between low and high MRG score (MRGS) groups. The 168 DEGs were shared by the above two DEGs gene sets (**Figure 1G**).

To gain insights into the biological functions associated with differentially expressed genes, we performed GO and KEGG enrichment analyses. The result of GO analysis revealed a predominant involvement of these genes in mitochondrial biology processes, including ATP biosynthetic process, cell growth, cellular respiration, electron transport chain, osteoblast differentiation, and oxidative phosphorylation (**Figure 1H**). The KEGG analysis demonstrated a close association between upregulated genes and oxidative phosphorylation, while downregulated genes were predominantly involved in signaling pathways, including PI3K-AKT and MAPK (**Figure 1I**).

### Consensus clustering of novel molecular subtype

**Figure 2:**
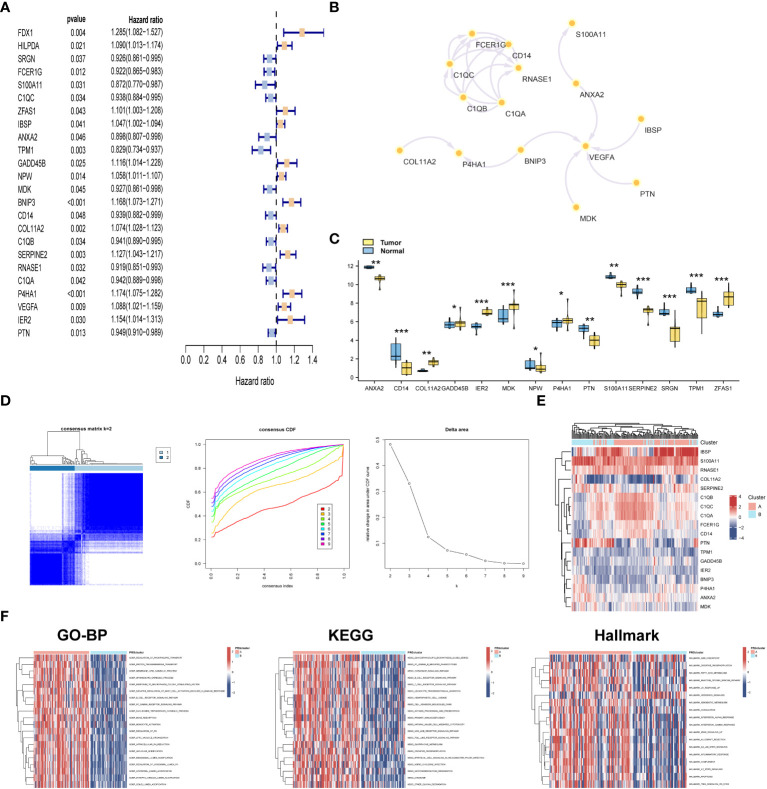
Consensus clustering of novel molecular subtype. **(A)** Univariate analysis for selecting prognostic genes. **(B)** PPI network of 24 prognostic genes. **(C)** Differential expression analysis of 24 prognostic genes. **(D)** Consensus matrices for k = 2 and cumulative distribution function (CDF) plot for 24 prognostic genes in OSA cases. **(E)** Heatmap of 24 prognostic genes in the two subtypes. **(F)** GSVA analysis of biological pathways among two subclusters. *p< 0.05, **p< 0.01, ***p< 0.001.

With the aim of detecting the prognostic value of 168 DEGs, univariate Cox analysis was conducted. The results indicated that a total of 24 DEGs were prognostically relevant in OSA (**Figure 2A**). Subsequently, we generated a PPI network based on these 24 genes (**Figure 2B**). More specifically, six genes demonstrated upregulated expression, whereas eight genes exhibited downregulated expression in malignant tissues (**Figure 2C**). Based on the expression levels of these 24 prognostic genes with differential expression, we performed a consensus clustering analysis to classify OSA samples. By setting the value of k to 2, which yielded the highest clustering stability, the combined OSA cohorts (TARGET cohort and GSE21257 cohort) were divided into two distinct molecular subtypes: cluster A (n = 85) and cluster B (n = 56) (**Figure 2D**). Significant distinctions in gene expression patterns between cluster A and cluster B were evident in the clustering heatmap, emphasizing the dissimilarities between the two clusters (**Figure 2E**). Moreover, the results of HALLMARK analysis exhibited substantial enrichment of pathways related to the G2M checkpoint, oxidative phosphorylation, fatty acid metabolism, and diverse inflammatory processes in the A subtype (**Figure 2F**).

### Construction of a prognostic model

**Figure 3:**
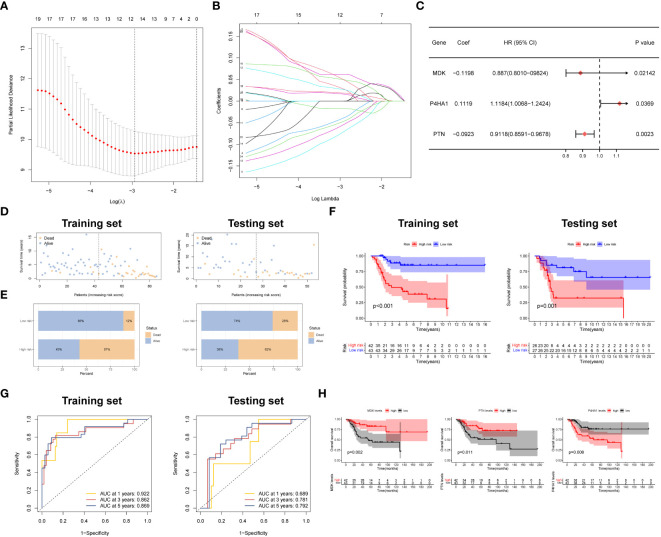
Development and verification of the prognostic signature. **(A, B)** LASSO regression with optimal lambda. **(C)** Stepwise multivariate Cox regression analysis. **(D, E)** Patient status distribution and Mortality rates of two groups. **(F)** Survival curves of OSA cases in two groups. **(G)** ROC curve of the prognostic signature. **(H)** Prognostic value of three model genes.

To develop a prognostic model, we conducted LASSO regression analysis on a panel of 24 prognostic DEGs in the training set (TARGET dataset), resulting in the identification of six candidate genes (**Figures 3A, B**). Subsequently, through the utilization of multi-Cox regression analysis, we ascertained three genes that independently served as prognostic factors, leading to the development of a reliable prognostic risk model (**Figure 3C**). The risk model equation was: (-0.1198 × MDK) + (0.1119 × P4HA1) + (-0.0923 × PTN). Employing the median risk value as a threshold, we segregated patients into two cohorts: the high-risk score group and the low-risk score group. Then, GSE21257 dataset was used to validate the performance of the model. Analysis of both the training and testing sets revealed a higher proportion of patient deaths within the high-risk score group in comparison to the low-risk score group, indicating a poorer prognosis in the former (**Figures 3D, E**). The Kaplan-Meier (K-M) survival curve indicated a substantially diminished clinical outcome in patients categorized under the high-risk score group when contrasted with those in the low-risk score group (**Figure 3F**).

To provide a more comprehensive evaluation of the prognostic significance of the risk model, time-dependent receiver operating characteristic (ROC) curves were constructed, and the corresponding area under the curve (AUC) was computed at different time points. **Figure 3G** presents the AUC values for the 1-year and 5-year survival rates in the training and testing groups. Specifically, the training group exhibited AUC values of 0.922 and 0.862, respectively, while the testing group displayed AUC values of 0.689 and 0.792 for the same survival rates.

To examine the impact of gene expression on patient prognosis, individuals were divided into high and low expression groups based on the levels of the three key genes, followed by K-M survival analysis. The results unveiled a noteworthy correlation between the expression levels of the three key genes and patient survival. Specifically, individuals with elevated expression of MDK and PTN displayed a significantly extended median overall survival (OS) compared to those with lower expression. Conversely, patients with reduced expression of P4HA1 demonstrated a significantly prolonged median OS relative to those with higher expression (**Figure 3H**). Also, we confirmed the expression patterns of MDK and P4HA1 in different OSA cell lines by PCR assay. The results demonstrated that MDK were lowly expressed in OSA cells, whereas P4HA1 was upregulated in OSA cells (**Supplementary Figure S1**).

### Evaluation of the relationship between risk model and clinical features

**Figure 4:**
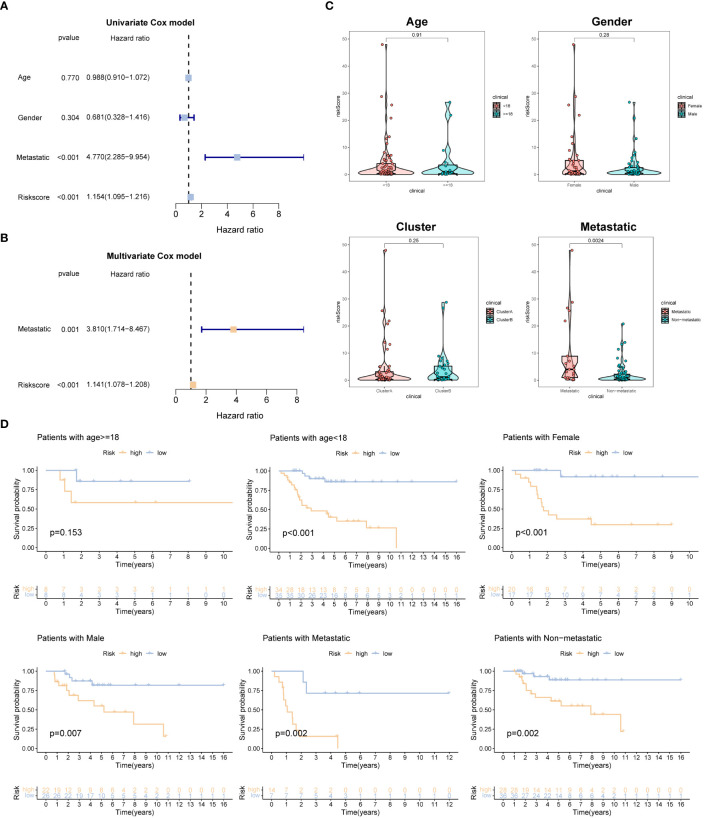
Independent prognosis analysis of the prognostic signature. **(A, B)** Univariate and multivariate Cox analyses for detecting independence of the signature. **(C)** The different levels of the risk regarding age, gender, molecular subtype and metastasis. **(D)** Survival analysis of different subgroups (age, gender, and metastasis).

To evaluate the association between the risk model and clinical features, we performed univariate and multivariate Cox regression analyses, incorporating various clinical characteristics, including age, gender, and metastatic status, along with the prognostic model. These analyses aimed to assess the independent prognostic value of the risk model while considering the potential confounding effects of these clinical factors. The findings consistently demonstrated that the risk model retained its statistical significance and independence as a prognostic factor for OSA cases (**Figures 4A, B**). These results further validate the clinical utility of the risk model in predicting the prognosis of OSA patients.

Additionally, we examined the disparities in risk scores among patients categorized into different subgroups based on clinical features. Interestingly, no significant differences in risk scores were observed when stratified by gender, age, and molecular subtype. However, among OSA patients with metastasis, the risk scores were higher compared to those without metastasis (**Figure 4C**). Furthermore, we grouped OSA patients based on age, gender, and metastasis status to explore the association between risk characteristics and prognosis within these clinical and pathological variables. Importantly, irrespective of age (<18 years old), gender (male, female), and presence of metastasis in patients with OSA, the low-risk group demonstrated significantly higher OS rates compared to the high-risk group (**Figure 4D**). These findings emphasize the efficacy of the risk score in predicting the prognosis of OSA cases within specific age groups and with different metastasis statuses.

### Single cell sequencing analysis

**Figure 5:**
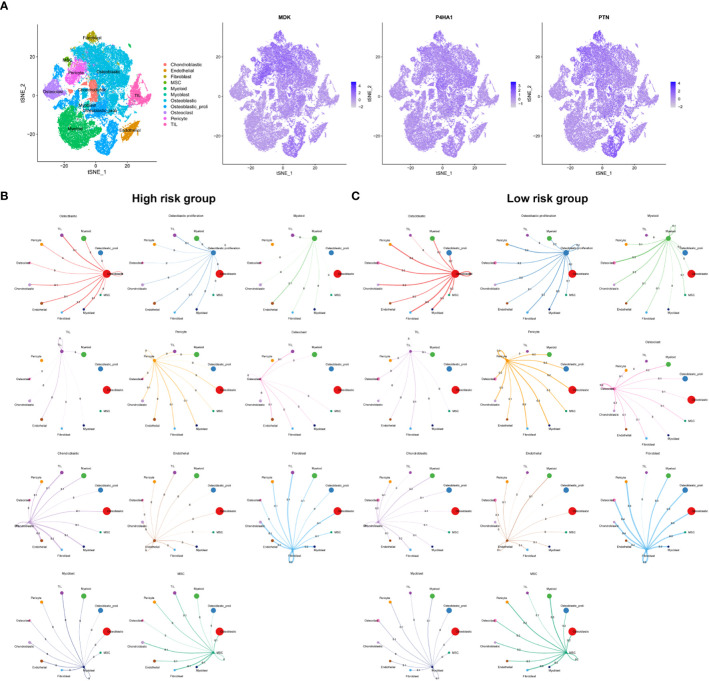
Single cell sequencing analysis. **(A)** Cellular location of three model genes. Circos plots show ligand-receptor interactions among different cells from the high-risk **(B)** and low-risk **(C)**. Branches connect pairs of interacting cell types and indicate the number of events in the graph.

To gain insights into the specific cell types and spatial regions associated with the three key prognostic genes related to OSA, further analysis was conducted. The findings indicated that these three genes were primarily expressed in pericyte cells and osteoblastic proliferative regions (**Figure 5A**). To explore the communication between different cells in the high and low-risk groups, we employed CellTalker, a tool that evaluates the expression of known ligand-receptor pairs within and between different cell populations. The outcomes of the cell interaction analysis unveiled a more pronounced occurrence of intercellular interactions among cells in the low-risk group, exhibiting a marked contrast to the high-risk group. These observations are visually represented in **Figures 5B, C**. This finding suggests that the disruption of harmonious cellular interactions in normal tissues contributes to the malignant progression and poor prognosis of tumors.

### Immune landscape of the risk signature

**Figure 6:**
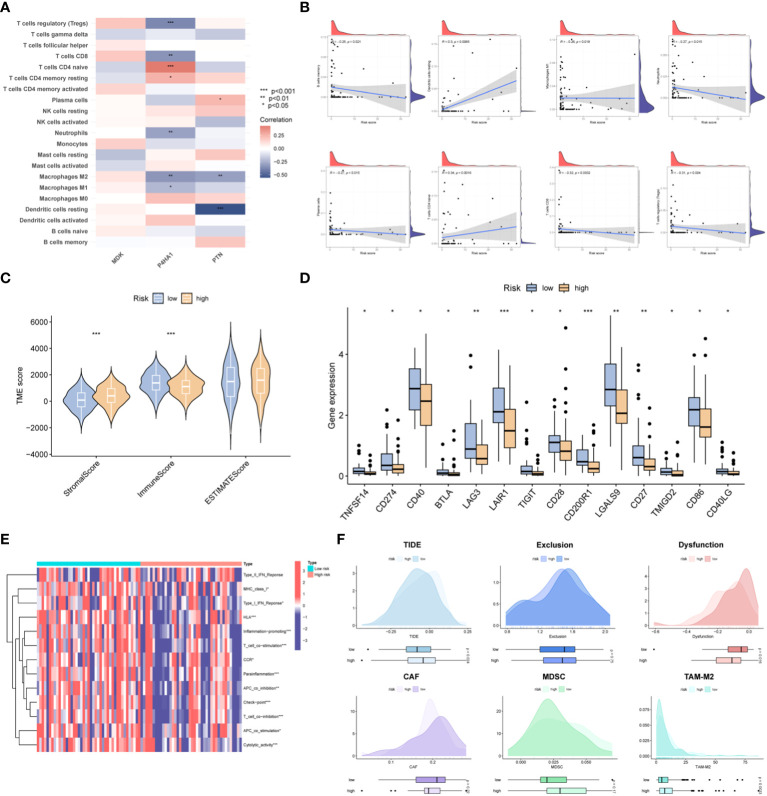
Immune landscape of the risk signature. **(A)** Heatmap demonstrating the association between three genes expression and infiltration level of different immunocytes. **(B)** Correlations between risk value and infiltration level of immune cells. **(C)** Violin plots depict the difference in stromal, immune and estimate scores between two groups. **(D)** Boxplots display the expression pattern of immune checkpoints among two groups. **(E)** GSVA analysis of immune functions between the two risk subgroups. **(F)** Prediction of ICB treatment response of OSA based on the TIDE, exclusion, dysfunction, CAF, MDSC and TAM-M2 scores. *p< 0.05, **p< 0.01, ***p< 0.001.

The dataset containing information on tumor-infiltrating immune cells was procured from the TIMER database. Spearman correlation analysis was employed to examine the correlation between crucial prognostic-related genes within the OSA tumor microenvironment (TME) and the abundance of immune cells. The analysis revealed that the expression of the P4HA1 gene was inversely correlated with the abundance of Tregs cells, CD8+ T cells, neutrophils, M1 and M2 macrophages. Conversely, a positive correlation was observed between the expression of the P4HA1 gene and the levels of CD4+ T cells and CD4+ T memory cells. Furthermore, a negative correlation was observed between the expression of the PTN gene and the abundance of dendritic cells and M2 macrophages, while a positive correlation was noted with the levels of plasma cells. Notably, there was no significant correlation between the expression of the MDK gene and immune cell levels (**Figure 6A**). The relationship between immune cell distributions and the risk score in OSA cases was investigated through correlation analysis. The results demonstrated that CD4+ T memory cells and dendritic cells demonstrated a positive association with the risk score, while the risk score showed an inverse correlation with the abundance of B cells, M1 macrophages, neutrophils, plasma cells, CD8+ T cells, and Treg cells in individuals with OSA cases (**Figure 6B**). The immune score was found to be lower and the stromal score higher in the high-risk group as compared to the low-risk group, as determined by the ESTIMATE analysis (**Figure 6C**). Further investigation was conducted to explore the correlation between the risk score and commonly used immune checkpoint inhibitors (ICIs) in tumors. The results indicated that higher risk scores were significantly associated with the downregulation of 14 ICIs, including TNFSF14, CD274, and CD40 etc. (**Figure 6D**). Analysis of immune cell function revealed that high-risk patients exhibited lower immune function (**Figure 6E**). Additionally, the TIDE framework was employed to evaluate the responsiveness of patients with different risk models to immunotherapy. The high-risk group exhibited significantly higher TIDE score and TAM-M2 score, along with a significant decrease in T-cell dysfunction, as indicated in **Figure 6F**.

### Clinical potency of the risk signature

**Figure 7:**
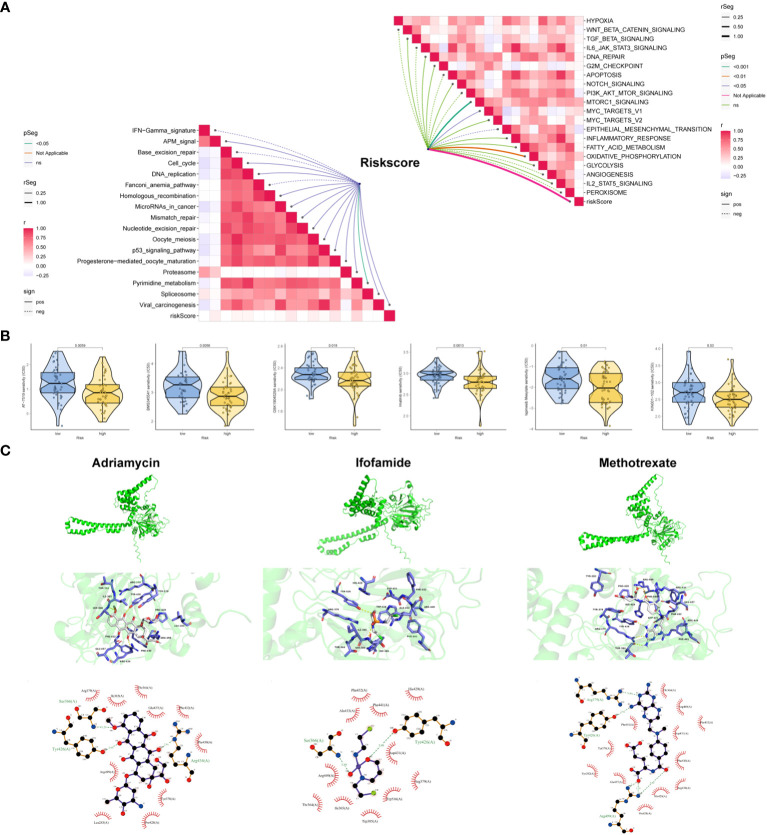
Clinical potency of the risk signature. **(A)** Correlation between the risk score and the hallmark (right) and immunotherapy prediction pathways (left). **(B)** Relationships between risk score and chemotherapeutic sensitivity of OSA. **(C)** Molecular docking depicts the docking position of the P4HA1 active pocket with Adriamycin, Ifosfamide, Methotrexate.

To gain deeper insights, we conducted further investigations into the association between risk scores for OSA cases and pivotal pathways previously implicated in OSA research, as well as signals linked to positive immune checkpoint blockade (ICB). The study findings revealed a noteworthy positive correlation between risk scores and key cancer-promoting pathways such as the MTORC1 signaling pathway, MYC TARGETS, and the oxidative phosphorylation signaling pathway. Conversely, the risk model demonstrated a negative correlation with epithelial-mesenchymal transition (EMT). In the analysis of immune-related pathways, a significant positive correlation was observed between risk scoring and various pathways including antigen processing and presentation machinery (APM) signaling, DNA replication, cell cycle, homologous recombination, microRNA, mismatch repair, and nucleotide excision repair, among others (**Figure 7A**). To facilitate clinical decision-making, we conducted an analysis to examine the association between cancer treatment drugs available in the GDSC database and risk scores. The results unveiled that patient classified in the low-risk group exhibited heightened sensitivity to six specific drugs (AT-7519, BMS345541, GSK1904529A, Imatinib, Ispinesib Mesylate, and KIN001-102). These findings provide evidence that risk score has the potential to predict the responsiveness of OSA patients to these treatment drugs (**Figure 7B**). Given that P4HA1 is a risk factor in OSA, molecular docking analysis was employed to identify the interaction structure between P4HA1 and three first-line chemotherapy drugs (Adriamycin, Ifosfamide, Methotrexate), which might can provide an important theoretical reference for targeting P4HA1 (**Figure 7C**).

### Determination of PTN as a novel suppressor in OSA

**Figure 8:**
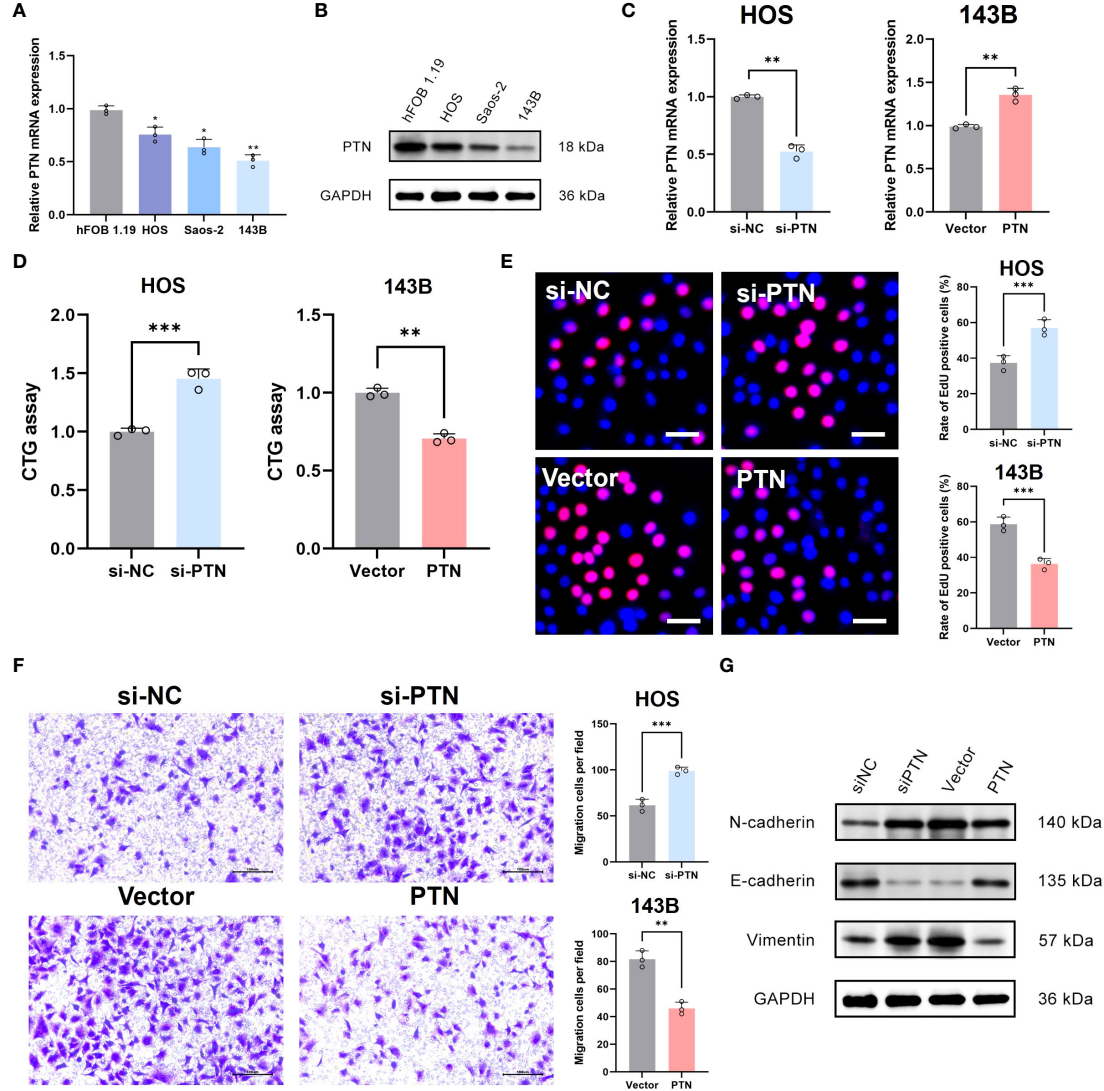
Determination of PTN as a novel suppressor in OSA. **(A, B)** The expression pattern of PTN at mRNA and protein levels in hFOB 1.19 and three OSA cell lines. **(C)** Transfection efficiency was tested by qRT-PCR. **(D, E)** The effect of PTN on cell viability was detected by CTG assay and EdU assay. Scale bar, 200 μm. **(F)** The effect of LCP1 on cell migration was detected using Transwell. Scale bar, 100 μm. **(G)** WB analysis investigate the relationship between PTN and EMT biomarker (N-cadherin, E-cadherin and Vimentin). *p< 0.05, **p< 0.01, ***p< 0.001.

PTN was chosen for the next experimental validation. We first observed that PTN was lowly expressed in OSA cell lines at mRNA and protein levels (**Figures 8A, B**). PCR assay exhibited good transfection efficiency of PTN in HOS and 143B cell lines (**Figure 8C**). To detect the proliferation of OSA cells, we conducted CTG assay and EdU assay. The results revealed that cell viability was greatly boosted in si-PTN group in HOS cells. (**Figures 8D, E**). Moreover, we found that silencing PTN could remarkably facilitate the migration ability of HOS cells (**Figure 8F**). The expression levels of N-cadherin and vimentin were upregulated in si-PTN group, whereas E-cadherin was downregulated (**Figure 8G**). Conversely, the opposite result was observed when PTN was overexpressed in 143B cells (**Figures 8D–G**).

## Discussion

Cuproptosis is a recently discovered form of RCD triggered by excess Cu^2+^. It is distinct from other cell death pathways, including apoptosis and necrosis (18). Current research provides insights into prospective clinical therapies via targeting cuproptosis. Mitochondria are a crucial therapeutic target for tumor since mitochondrial dysfunction could trigger cellular oxidative stress leading to cell death (19). A recent study proposes that cuproptosis is highly related to mitochondrial metabolism and respiration (20). However, the potential molecular interactions that link copper ion-mediated cell death, and mitochondria in OSA are still elusive.

The present project successfully generated a prognostic risk model in OSA based on cuproptosis and mitochondria-associated signatures. Midkine (MDK) a heparin-binding growth factor initially identified as a production of retinoid-responsive genes in the embryonic formation process. MDK expression was found to be upregulated in numerous human neoplasms (21). It acted as a cancer driver, facilitating tumor cell proliferation, survival and metastasis. A number of investigations have reported the ability of A to act as a prognostic marker for tumors and as a target for management of tumors. Furthermore, MDK also could boost therapeutic resistance and immune regulation in the TME (22). MDK can facilitate tumor development through activation of cancer signaling pathways mediated by receptor-ligand interactions. As reported by Xia et al., MDK could boost tumor growth and survival by binding with LKB1 which in turn blocks activation of AMPK (23).

Polytrophic factor (PTN) is a secreted heparin-binding growth factor exhibiting important regulatory effects on tumors. As a small cationic protein, PTN is involved in a variety of biological processes including cancer cell growth and metastasis (24). PTN and its receptor RPTPβ are unregulated in various tumors. Functional experiments have revealed that this receptor ligand pair can modulate the proliferation and migration ability of cancer cells (25). In addition, M2-like macrophages could facilitate the malignant behavior of brain tumor by the interaction of PTN and PTPRZ1 (26).

P4HA1 gene contains a subset of proline 4-hydroxylase, which is a pivotal enzyme in collagen synthesis consisting of two identical alpha and beta subunits (27). P4HA1 has been found to exert a carcinogenic effect. In esophageal cancer, activation of P4HA1 by STAT1 transcription could boost cell growth and survival (28, 29). Zhou et al. demonstrated that elevated expression of P4HA1 indicates dismal prognosis in cases with head and neck cancer (HNC). P4HA1 could confer ferroptosis resistance to HNC cells through activation of HMGCS1, suggesting P4HA1 could be a promising target for the HNC management (30). As revealed by Eriksson et al., inhibition of P4HA1 expression could inhibited melanoma tumor metastasis. Mechanistically, silencing A decreases collagen deposition in the basement membrane of tumor vessels, contributing to vessel wall rupture and hemorrhage (31).

Next, immunocytic infiltration analysis indicated that risk value was negatively correlated with the level of infiltration of T cells and M1 macrophages cells. Previous studies have demonstrated that activated CD8+ T cells potently suppressed OSA proliferation. Moreover, CD8+ T cells had a positive correlation with a favorable prognosis in OSA cases. Tang et al. indicated that OSA cells with elevated levels of T synthase facilitate growth of CD8+ T cells and block apoptosis to enhance tumor lethality (32). Macrophages in TME are defined as tumor-associated macrophages (TAM). Such plastic cells are subdivided into anti-cancer (M1-like macrophages) and pro-cancer types (M2-like macrophages). M1-like TAMs repressed OSA cells survival on activation with IFN-γ. On the contrary, higher infiltration of M2-type TAMs boots OSA migration and invasion and serves as a marker of dismal prognosis of OSA cases (33).

Type I interferon (IFN) response plays a central part in human immune surveillance. Type I IFN signaling enables full efficacy of various anti-cancer drugs, including chemotherapeutic agents. In addition, upregulation of IFN-stimulated gene (ISG) expression points to a favorable prognosis for patients with several cancers, including melanoma and breast cancer. Recombinant type I IFNs have been successfully utilized for the therapy of various human tumors (34, 35). Our data suggested that IFN response is remarkably enriched in the low-risk group, which confirmed OSA patients with low-risk value have a better prognosis.

Immunotherapy has played an important role in the treatment of solid tumours in recent years (36). Therefore, we further analyzed the likelihood of immune escape in patients by TIDE to assess whether the patients could benefit from immunotherapy. TIDE is an algorithm to assess the potentials of tumor immune escape based on mRNA expression data of tumor cases. This method can also estimate T-cell dysfunction and immunotherapy resistance based on extensive clinical data (37, 38). Our results demonstrated that patients in the high-risk group have an increased TIDE score, implying that this group is not as sensitive to ICI treatment.

There were several shortcomings in the present research. First, our nominated was developed by public databases; therefore, additional large-scale prospective and multicenter clinical studies are warranted to validate our data. The role of PTN in OSA was only detected by *in vitro* experiments. Further *in vivo* or molecular experiments are needed in the future to demonstrate the role of PTN.

## Conclusion

In short, cuproptosis and mitochondria-associated signatures with significant prognosis value can differentiate between molecular subgroups of OSA. A robust risk model based on signature could evaluate OSA prognosis. Furthermore, the risk score can mirror tumor immune landscape and predict efficacy of chemotherapy for OSA patients.

## Data availability statement

The datasets presented in this study can be found in online repositories. The names of the repository/repositories and accession number(s) can be found in the article/**Supplementary Material**.

## Ethics statement

Ethical approval was not required for the studies on humans in accordance with the local legislation and institutional requirements because only commercially available established cell lines were used.

## Author contributions

CJ: Investigation, Methodology, Writing – original draft. FZ: Software, Visualization, Writing – review & editing. ML: Writing – review & editing, Data curation, Validation. LY: Writing – review & editing, Software, Supervision. SL: Data curation, Methodology, Visualization, Writing – review & editing. ZL: Writing – review & editing, Data curation. YH: Writing – review & editing, Conceptualization, Resources.
