# Supplementary material for: Multi-omics analysis reveals cuproptosis and mitochondria-based signature for assessing prognosis and immune landscape in osteosarcoma
Source: Front Immunol. 2024 Jan 5;14:1280945. doi: 10.3389/fimmu.2023.1280945 (PMC10796547; doi:10.3389/fimmu.2023.1280945)
Supplement: Supplementary file 1 [file DataSheet_1.docx]

https://www.jianguoyun.com/p/DWfguxoQzc6eChiSn5UFIAA
