# Supplementary material for: Multi-omics analysis reveals cuproptosis and mitochondria-based signature for assessing prognosis and immune landscape in osteosarcoma
Source: Front Immunol. 2024 Jan 5;14:1280945. doi: 10.3389/fimmu.2023.1280945 (PMC10796547; doi:10.3389/fimmu.2023.1280945)
Supplement: Supplementary file 2 [file DataSheet_2.docx]

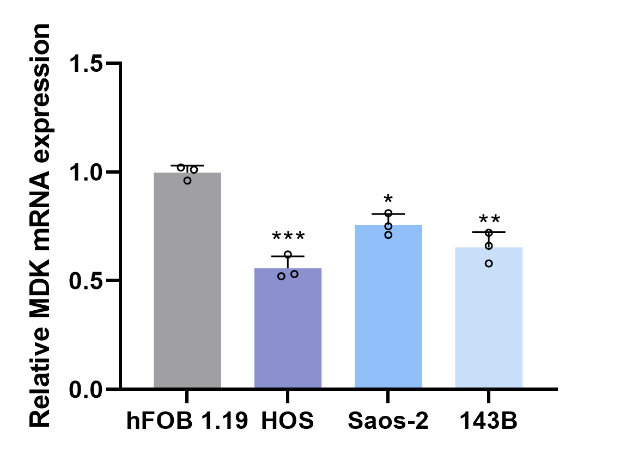

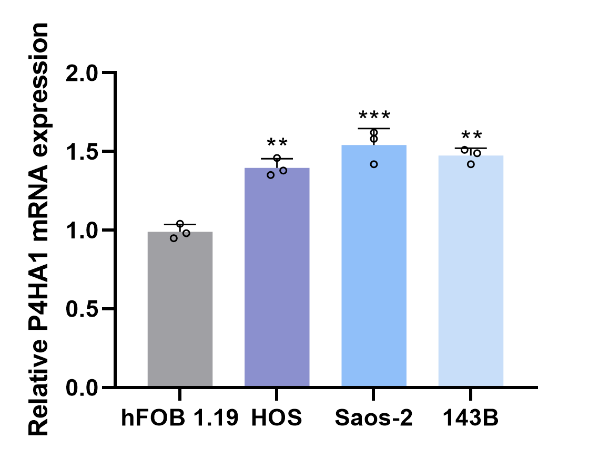


**Figure S1: The expression patterns of MDK and P4HA1 were verified by in different OSA cell lines by PCR assay.**
