# Supplementary material for: Multi-omics analysis reveals cuproptosis and mitochondria-based signature for assessing prognosis and immune landscape in osteosarcoma
Source: Front Immunol. 2024 Jan 5;14:1280945. doi: 10.3389/fimmu.2023.1280945 (PMC10796547; doi:10.3389/fimmu.2023.1280945)
Supplement: Supplementary file 3 [file Table_1.docx]

**Supplementary Table 1. Primer and siRNA sequences for each gene.**

| Gene | Primer sequence | |
| --- | --- | --- |
| GAPDH | Forward | 5′-CTGGGCTACACTGAGCACC-3′ |
|  | Reverse | 5′-AAGTGGTCGTTGAGGGCAATG-3′ |
| PTN | Forward | 5′-GGAGCTGAGTGCAAGCAAAC-3′ |
|  | Reverse | 5′-CTCGCTTCAGACTTCCAGTTC-3' |
| Gene | siRNA-PTN sequence | |
| siRNA | 5′-AGGCAAGAAACAGGAGAAGAT-3′ | |
